# Transformation of Muscular Fibre into Fat

**Published:** 1865-06

**Authors:** 


### TRANSFORMATION OF MUSCULAR FIBRE INTO FAT.

At a recent meeting of the Boston Society for Medical Improvement, Dr. J. Wyman stated that he had seen fatty degeneration occur in muscular fibre under the following circumstances: Portions of muscle were introduced into a flask with water, thoroughly boiled, supplied with air through a tube heated to redness, and then the flask was hermetically sealed. In the course of three or four weeks, on examination with the microscope, the fibres, without having become putrid were found to have the sarcolemma entire; in some the fibrillæ were still distinct, in others they were partially replaced by granules, and in others they had wholly disappeared and the fibre sheath was filled with globules of oil.

Dr. Ellis had examined some of the specimens and recognized the same appearances which are seen in ordinary cases of fatty degeneration. Rudolph Wagner found that various albuminoid substances underwent a similar change when introduced into the abdominal cavity of a living animal. It was objected to his experiments that the fat may have been secreted by the animal and substituted for the materials of the tissue introduced, these having disappeared. In the experiments here given, such an explanation is inapplicable, for since the muscular fibre was only surrounded by water, there could be no other source for the fat than the fibre itself.

In view of these facts it seems probable, as Fourcroy originally suspected, that adipocire can be derived from muscle which has undergone transformation into fat.—*Boston Med. Journal.*

### INFLUENCE OF A LONG COURSE OF NITRIC ACID IN REDUCING THE ENLARGEMENT OF THE LIVER AND SPLEEN THAT SOMETIMES RESULTS FROM THE SYPHILITIC CACHEXY.

The enlargement which Dr. Budd refers to, is that which has been latterly described as due to amyloid degeneration. The most striking examples of it are seen in the victims of scrofulous or syphilitic caries. Three cases are related in which mxx of dilute nitric acid were taken ter die for a period varying from fifteen to four months, without inducing excessive acidity of the urine or any inconvenience attributable to undue acidity of the stomach. Sarsaparilla, iron, or bark, were conjoined with the acid. The result of his experience leads Budd to conclude that when the liver and spleen have become diseased in the manner specified, in sequel to protracted syphilitic disease of the bones, nitric acid, long taken, has a remarkable influence in gradually effecting the removal of the morbid deposit to which these organs owe their increased size, restoring the organs to a more healthy condition, and improving the general health. The cases further afford a strong presumption that nitric acid, taken earlier, would prevent the disease of the abdominal glands, which, when established, it tends to remedy. It is, however, essential that the disease of the bone, on which the enlargement of the liver and spleen is consequent, should be arrested; if this cannot be effected, the malady, though even then its course may be retarded, usually makes progress, and life is cut short by renal disease, which very often accompanies that of the liver and spleen. Budd suggests that a long course of nitric acid may have influence in remedying and preventing glandular enlargements, chronic ulcers, and other forms of scrofulous disease. He is persuaded that in tuberculous disease of the lung, nitro-muriatic acid, long taken, tends to prevent the further deposit of tubercle.—*Sydenham Society's Year-Book*, 1863, from *Brit. Med. Journ.*

